# Effects of self-management intervention on health outcomes of patients with heart failure: a systematic review of randomized controlled trials

**DOI:** 10.1186/1471-2261-6-43

**Published:** 2006-11-02

**Authors:** Aleksandra Jovicic, Jayna M Holroyd-Leduc, Sharon E Straus

**Affiliations:** 1 Department of Mechanical and Industrial Engineering, University of Toronto, 5 King's College Road, Toronto, Ontario, M5S 3G8, Canada; 2 Knowledge Translation Program, Faculty of Medicine, University of Toronto, 500 University Avenue, Suite 300, Toronto, Ontario, M5G 1V7, Canada; 3 St. Michael's Hospital, University Health Network, Toronto, Ontario, Canada; 4 Department of Medicine, University of Calgary, Calgary, Alberta, Canada

## Abstract

**Background:**

Heart failure is the most common cause of hospitalization among adults over 65. Over 60% of patients die within 10 years of first onset of symptoms. The objective of this study is to determine the effectiveness of self-management interventions on hospital readmission rates, mortality, and health-related quality of life in patients diagnosed with heart failure.

**Methods:**

The study is a systematic review of randomized controlled trials. The following data sources were used: MEDLINE (1966-11/2005), EMBASE (1980-11/2005), CINAHL (1982-11/2005), the ACP Journal Club database (to 11/2005), the Cochrane Central Trial Registry and the Cochrane Database of Systematic Reviews (to 11/2005); article reference lists; and experts in the field. We included randomized controlled trials of self-management interventions that enrolled patients 18 years of age or older who were diagnosed with heart failure. The primary outcomes of interest were all-cause hospital readmissions, hospital readmissions due to heart failure, and mortality. Secondary outcomes were compliance with treatment and quality of life scores. Three reviewers independently assessed the quality of each study and abstracted the results. For each included study, we computed the pooled odds ratios (OR) for all-cause hospital readmission, hospital readmission due to heart failure, and death. We used a fixed effects model to quantitatively synthesize results. We were not able to pool effects on health-related quality of life and measures of compliance with treatment, but we summarized the findings from the relevant studies. We also summarized the reported cost savings.

**Results:**

From 671 citations that were identified, 6 randomized trials with 857 patients were included in the review. Self-management decreased all-cause hospital readmissions (OR 0.59; 95% confidence interval (CI) 0.44 to 0.80, P = 0.001) and heart failure readmissions (OR 0.44; 95% CI 0.27 to 0.71, P = 0.001). The effect on mortality was not significant (OR = 0.93; 95% CI 0.57 to 1.51, P = 0.76). Adherence to prescribed medical advice improved, but there was no significant difference in functional capabilities, symptom status and quality of life. The reported savings ranged from $1300 to $7515 per patient per year.

**Conclusion:**

Self-management programs targeted for patients with heart failure decrease overall hospital readmissions and readmissions for heart failure.

## Background

It is estimated that as many as 30 million of the one billion people living in the 47 nations represented by the European Society of Cardiology are living with heart failure (HF) [1]. The estimated number of afflicted in North America exceeds five million [2]. Heart failure is characterized by high mortality and hospitalization rates, physical, emotional and functional impairment, reduced quality of life, and increased caregiver burden [3,4]. Heart failure is the most common cause of hospitalization for adults over the age of 65 years [2]. Approximately 59% of men and 45% of women die within five years of the first onset of the symptoms of the illness [5].

Disease management programs can reduce hospitalization and may improve the quality of life of patients diagnosed with heart failure [6-10]. Complex case management interventions demonstrated positive effects on mortality, quality of life, and length of hospitalization [10]. These results were likely achieved through integrated and comprehensive programs that include detailed assessment of patients, optimization of medications, patient education, and frequent monitoring by medical professionals. Less is known about the effectiveness of self-management strategies on the risk of hospital readmission and mortality for patients with heart failure. Unlike disease management and case management programs, self-management programs aim to enable patients to assume primary role in managing their condition: monitor symptoms, adjust medications and determine when additional medical attention is necessary. These interventions have been receiving much attention in recent years because of the potential to reduce resource utilization while improving patient health outcomes.

Self-management strategies have proven beneficial in chronic diseases such as asthma and type 2 diabetes mellitus [11,12], but their effects on other conditions including chronic obstructive airways disease are unclear [11,13]. Given the incidence and severity of heart failure, it is important to understand the impact of self-management strategies on clinical outcomes. This review evaluates the effectiveness of self-management strategies on hospital readmission, death and quality of life in patients with heart failure.

## Methods

### Searching

We searched MEDLINE from 1966 to November 2005, EMBASE from 1980 to November 2005, CINAHL from 1982 to November 2005, the ACP Journal Club database, the Cochrane Central Trial Registry and the Cochrane Database of Systematic Reviews. The search strategy used the terms: "self management", "self administration", "self medication", "quality of care", "quality of healthcare", "disease management", "congestive heart failure" and "heart failure, congestive". We limited the studies to randomized controlled trials using the terms "randomized controlled trial", "controlled clinical trial", "random allocation" and "random*". We placed no restrictions on languages. We identified additional articles from reviewing the bibliographies of retrieved articles and from consulting experts in the field. Further details on the search strategies are available from the authors.

### Selection

We included randomized controlled trials of self-management interventions that enrolled patients 18 years of age or older who were diagnosed with heart failure. The target population consisted of patients hospitalized for heart failure who were enrolled in self-management programs during the hospitalization or at discharge.

Health Canada defines self-management as "decisions and actions taken by someone who is facing a health problem or issue in order to cope with it and improve his or her health" [14]. The operational definition of self-management interventions included programs aimed at enabling patients to assume responsibility for managing one or more aspects of heart failure (e.g. symptom monitoring, weight monitoring, medication dosage adjustment and/or decision-making). To be included in the review, the intervention had to be a self-management intervention in which patients retained the primary role in managing their health condition. We excluded interventions with self-management components in which physicians or nurses were involved in medical assessment or therapy optimization, because their involvement interfered with the patients' role as primary decision-makers in managing their own care. We excluded educational interventions from consideration, unless they explicitly declared self-management to be the primary objective of the intervention. We placed no restriction on the method of communication exchange or education (in person, telephone, email, written, verbal, visual, electronic or audio).

The primary outcomes of interest were all-cause hospital readmissions, hospital readmissions due to heart failure and mortality. Secondary outcomes were compliance with treatment, adherence to self-management strategies and quality of life scores. We included studies that collected data relevant to the primary and secondary outcomes, regardless of the measure of hospital readmission. Since we were primarily interested in determining the effects of the intervention on readmission to hospital and mortality, we excluded studies that enrolled the patients in the community setting.

### Validity assessment

Two reviewers (SJ, SES) independently reviewed the abstracts obtained in the search and retrieved the full text article of those that met the inclusion criteria. In cases of disagreement, we retrieved the full text article for review. Three reviewers (SJ, JHL, SES) independently reviewed all retrieved articles to confirm that the inclusion criteria were met and to assess the quality of each study. The quality assessment criteria included the method of randomization, details of allocation concealment, blinding of health care provider, outcome assessors and data analysts, intention-to-treat analysis and method of outcome assessment. The reviewers resolved differences in assessment through discussion to achieve consensus.

### Data abstraction

Three reviewers (SJ, JHL, SES) independently extracted data on the details of the intervention and the outcomes of interest from all studies selected for inclusion in the systematic review. The reviewers resolved differences in assessment through discussion to achieve consensus.

### Quantitative data synthesis

For each study, the authors computed the pooled odds ratios (OR) for dichotomous variables including all-cause hospital readmission, heart failure readmission and death of patients in the intervention and control groups. Statistical heterogeneity was assessed using the Q-test [15]. A fixed effects model based on the Mantel-Haenszel [16] test was used for quantitative data synthesis. Data was synthesized using Comprehensive Meta Analysis software [17]. Since different variables and measures were used to assess quality of life, we were unable to effectively pool quality of life scores, but we described trends in the quality of life and health behaviors such as adherence to medical advice and prescribed self-management behaviors.

## Results

### Trial flow

The search strategies yielded 642 articles. We identified an additional 29 articles from personal files, reference lists of retrieved articles and from communication with experts. Based on two independent reviews of the abstracts, we retrieved the full text of 65 articles. The agreement on the reviewers' initial assessment on whether to include or exclude the articles these articles was 89% (58 articles of the 65 articles retrieved). After excluding articles that did not meet the inclusion criteria and removing duplicates, we identified six studies of sufficient quality for inclusion in the systematic review. All excluded studies, except for one, were excluded because they either did not meet the definition of self-management or because their results were published in more than one study. One self-management study was excluded because it was conducted in a community setting.

### Study characteristics

Table 1 summarizes key characteristics of the selected studies. The sample sizes in included studies ranged from 70 [18] to 223 [19]. The quality of the studies varied. Some studies did not clearly specify whether patient allocation was concealed [21] or whether outcome assessors and data analysts were blinded to treatment [22]. The mean age of the participants in the studies ranged from 56 to 76 years of age [18,23], with 24% to 47% females [22,23]. Statistics on race were not reported consistently. In the studies that provided details on race, participants were described as predominantly white [20,23] or not black [19]. Three studies reported information about level of patient education. The participants in the study by Sethares et al had a mean education level of grade 11 and approximately 72% of participants in the study by Cline et al. had primary school education [18,22]. The study by Ross et al., included a disproportionate number of white, college-educated males earning $45,000 or more [23].

**Table 1:** Study characteristics. Characteristics of the studies included in the systematic review of self-management interventions

**Citation**	**Intervention**	**Study Population Total (Control/Intervention)**	**Follow-up & Assessment method**	**Outcomes**
Cline et al. (Sweden)	Education on heart failure for patients and their families. Guidelines for self-management of diuretics based on signs and symptoms and instructions on when to contact the nurse. Provision of 7-day medication organizer. Nurse counselling: 2 × 30 min during hospitalization, 1 × 1 hr after discharge	N = 190 (110/80) Male: 53% Mean age: 75.6 Age range: 65–84 Mean NYHA: 2.6	1 year; Self-administered questionnaires, hospital records	Readmission, hospitalization days, health care costs during one year, quality of life, mortality
Jaarsma et al. (Netherlands)	Education about consequences of heart failure and guidelines for compliance, fluid balance, recognition of warning symptoms. Counselling: Average of 4 sessions during hospitalization, 1 phone call & 1 home visit after discharge NOTE: control group received education about medication and lifestyle	N = 179 (95/84) Male: 58% Mean age: 73. NYHA III: 17% NYHA III-IV: 21%, NYHA IV: 61%.	1, 3, 9 months; Patient interviews, questionnaires	Self-care abilities, self-care behaviour, quality of life, overall wellbeing, readmission, hospitalization days, resource utilization.
Koelling et al. (U.S.)	Education (1 hour), provision of instructions for taking medications, weighing, dietary restrictions & symptom monitoring, including when to contact physicians	N = 223 (116/107) Male: 58% Mean age 65: Black: 22% Coronary disease 64%	6 months; Phone call from nurse at 1, 3 and 6 months	Readmission (heart failure, cardiac and all-cause), mortality, cost of care, self-practice scores.
Krumholz et al. (U.S.)	Education about illness, medication, early signs & symptoms, health behaviour, when to seek help. Weekly phone call for 4 weeks, biweekly for 8 weeks, monthly for the remainder of the year	N = 88 (44/44) Male: 66/48% Mean age: 71.6/75.9 White: 77/70%	1 Year; Review of records, next of kin contact, discharge information	Readmission (heart failure, cardiovascular disease and all-cause), hospitalization days, mortality, cost of care.
Ross et al. (U.S.)	Educational software, a messaging system enabling communication between patients and staff	N = 107 (53/54) Male: 74/80% Mean age: 55/57 College: 44/53% (v. decliners: 26%) White: 88/92% (v. 75% decliners) Household income >$45 K: 50/56% (v. 76% decliners).	6 months, 1 Year; Mailed survey	Readmission, mortality, health status, self-efficacy, adherence to medical advice and patient satisfaction.
Sethares & Elliot (U.S.)	Nurse-led tailored message intervention based on perceived benefits and barriers to self-care of HF. Follow-up 1 week and 1 month after discharge NOTE: Patients in the control group were given information about medication and possibly referred to nurse agencies	N = 70 (37/33) Mean Age: 76.8/75.7 Mean NYHA: 3.0 Education (years): 11/11	3 months; Health-measure scales	Readmission, quality of life, beliefs in benefits and barriers of treatment.

### Interventions

All studies but one [23] consisted of education and limited follow-up (Table 1). In all studies, the patients were taught to monitor their condition and to recognize when to seek medical assistance. Education typically included information about signs and symptoms of heart failure, importance of daily weighing, dietary restrictions and of adherence to the medication regimen. The education sessions in the study by Krumholz et al. differed from others in that they strongly focused on inter-relationships between diet, medication, illness, and health behavior [20]. By contrast, the patients in the study by Ross et al. were given only educational software with information about heart failure and with tools for self-monitoring [23]. Patients in this study were able to communicate with nursing staff through a messaging system integrated with the software, but no other follow up was part of this study. All studies, except for the one by Krumholz, involved limited follow-up. In the study by Krumholz, patients received telephone calls of decreasing frequency, ranging from once weekly to once monthly.

### Outcomes

Length of follow-up varied across the trials, ranging from three to twelve months. All studies reported rate of hospital readmission as a primary or secondary outcome. Four of six studies reported mortality rates at one year. Three of six studies reported scores for patients' quality of life. Three studies reported effects on patients' health behavior.

### All-cause readmission

**Figure 1:**
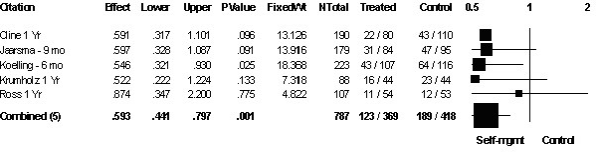
**Patients readmitted to hospital**. Patients readmitted to hospital for all reasons during the first year after discharge from hospital.

Five studies [19-23] with 787 patients reported the number of readmissions during the one year follow-up period (Figure 1). No statistically significant heterogeneity was found amongst these studies (Q-test (4DF) = 0.856, *P *= 0.93). Pooling of these results identified a significant decrease in hospitalizations with self-management (OR 0.59; 95% CI 0.44 to 0.80). The effect remained unchanged after the study that did not clearly specify whether patient allocation was concealed [21] was removed from the analysis (OR 0.59; 95% CI 0.42 to 0.83). A funnel plot showed no evidence of publication bias.

### Readmissions due to heart failure

**Figure 2:**
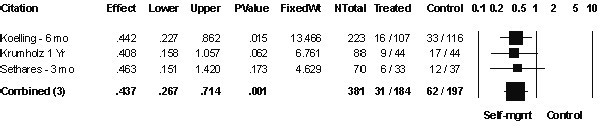
**Patients readmitted for heart failure**. Patients readmitted to hospital for heart failure within 3 or more months after discharge from hospital.

Three studies [18-20] with a total of 381 patients reported data on the number of patients readmitted for heart failure (Figure 2). There was no significant heterogeneity amongst these trials (Q-test (2DF) =.031, *P *= 0.98). Self-management strategies decreased the risk of readmission due to heart failure (OR 0.44; 95% CI 0.27 to 0.71). A funnel plot showed no evidence of publication bias.

### Mortality

**Figure 3:**
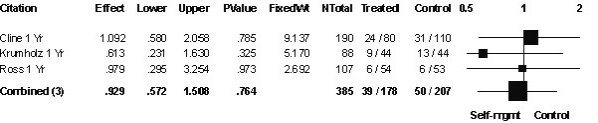
**Patient mortality**. Patient mortality rates at 1 year after hospital discharge.

Three of six studies reported mortality rates at one year (Figure 3) [20,22,23]. The CochranQ_2 statistic for heterogeneity was not significant (Q (2DF) = 0.95, *P *= 0.76). There was no significant reduction in mortality with self-management (OR 0.93; 95% CI 0.57 to 1.51) (Figure 3). Funnel plot showed no evidence of publication bias.

### Quality of life scores

Three studies [18,21,22] included measurements of quality of life. Since each study used a different scale, we could not effectively pool the overall data. The studies consistently reported no significant improvements in quality of life scores. Sethares et al reported no difference in quality of life compared to standard care as measured by the Minnesota Living with Heart Failure Questionnaire [18]. Jaarsma and colleagues found no statistically significant differences in functional capabilities and symptom status (occurrence, severity and distress) at 3 and 9 months after discharge, as measured by the Appraisal of Self-care Agency Scale [22]. Cline and colleagues reported no difference in quality of life as measured by a combination of other questionnaires, including the Nottingham Health Profile [22].

### Health behavior

Improvements in compliance with treatment and prescribed health behaviors were reported in two studies [19,23]. In the study by Koelling [19], the patients in the intervention group were significantly more likely to have an action plan, perform daily weighing, monitor symptoms, adhere to sodium and fluid restriction, exercise, and not smoke. The study also reported decreased beliefs in barriers and increased beliefs in benefits of diet and self-monitoring [19]. Ross et al. reported a significant improvement in adherence to general medical advice, and a non-significant trend toward improvement in self-efficacy and medication administration [23].

### Cost

Cost-effectiveness analysis could not be completed due to inherent challenges in comparing costs among studies conducted at different time periods and across different health-care systems. However, all studies that examined cost-effectiveness reported that self-management interventions resulted in savings compared to standard care, due to reduced resource utilization [19,20,22]. Krumholz et al., Cline et al. and Koelling et al. reported that, after subtracting the cost of the intervention, US$7515, US$1300 and US$2823 respectively were saved per patient in one year. Greater savings were achieved in the two U.S. studies than in the Swedish study. As expected, the greatest cost savings were achieved in the study that demonstrated greatest decrease in readmission.

## Discussion

This systematic review demonstrates that a self-management intervention in patients with heart failure decreases hospital readmissions, both all-cause and HF-related. There was no significant effect on mortality or on health related quality of life. However, health behaviors, such as regular weighing and monitoring of symptoms, increased in patients who were enrolled in self-management programs. The decrease in all-cause readmission may be partly due to the decrease in HF-readmissions. Also, as self-management interventions in this study encouraged beneficial health practices, these interventions may have had the auxiliary effect of reducing readmissions for causes other than heart failure.

Results of the individual studies seem to indicate that the self-management interventions with more intensive education components, such as that in Krumholz et al, are more likely to show benefits than those with less intensive education sessions. In the study which showed the least benefit in reducing all-cause readmission [23], the patients were given educational software and a messaging system but were not otherwise involved in structured follow-up or nurse-led education. In this study, the readmission rates in the intervention group did not significantly differ from those in the usual care group. The studies also involved different degree of contact with medical staff. It is difficult to assess from the available data to what degree, if any, communication frequency during follow up affected the primary outcomes, because the studies that involved more frequent follow up also involved more education. Moreover, it is not clear to whether the follow-up by medical professionals affected patients' health status. Although we excluded the studies in which doctors or nurses assessed patients' health or altered their health regimen, it is possible that contact with medical professionals influenced patients' health outcomes. Further research is needed to conclusively determine the effects of the quantity of self-management education, the method of delivery, and the duration and nature of follow up on health outcomes.

The results of the study by Ross et al. indicate that patients who communicated with medical staff through messaging software were as satisfied with the communication as were the patients undergoing standard care [23]. These results must be interpreted with caution, since nearly all the participants in this study had access to a computer at home and had previous experience using the Internet, compared to about 50% of those who declined to participate. Future studies should examine the effectiveness of electronic communication in broader patient populations.

This study adds to the current body of literature in that it analyses the effect of self-management interventions on health outcomes of patients with heart failure. The findings of this review are consistent with results from systematic reviews of disease management strategies for patients with heart failure. Multi-disciplinary heart failure management programs that involve specialized follow-up significantly decreased hospital readmission but did not affect mortality rates [6,8]. Similarly, a systematic review reported that telemonitoring interventions reported may decrease the number of readmissions as well as reduce mortality and morbidity [25]. As in the case of the self-management interventions, multidisciplinary heart failure management programs and telemonitoring programs can include several components, and it is difficult to evaluate to what extent they contribute to the overall effect on patients' health.

As with any systematic review, the strength of the results and the extent of the analysis that is possible depend on the strength of and the data reported in the individual studies. Results for heart failure and mortality were each reported in three of the six studies, and thus data was available for less than one half of the patients in the included studies, which may have affected the strength of the results. And, not all studies described details about blinding [22]. Moreover, the included studies did not consistently report statistics on race or socioeconomic factors such as the level of education. Social conditions such as level of education have been found to be more powerful predictors of health status than many of the risk factors associated with cardiovascular disease [24]. Insufficient data was also provided for other predictors such as ethnicity and smoking [24]. Unavailability of such information could influence the results of individual studies as well as the combined result. In the study by Jaarsma et al., the patients who dropped out were significantly older, more frequently lived in the nursing home, were diagnosed with hypertension, and had cardiomyopathy as the underlying cause of heart failure. Differences in attrition between the control and intervention group could influence the results of this study [20].

## Conclusion

The results of this meta-analysis indicate that a self-management program for patients with heart failure decreases both all-cause hospital readmissions and readmissions due to heart failure. The effect on mortality and quality of life is inconclusive based on the current body of evidence, though improvements in health behavior were demonstrated. Future research is needed to assess whether improvements in mortality and quality of life can be achieved with self-management and to determine what components of self-management are necessary to improve clinical outcomes.

## Competing interests

The author(s) declare that they have no competing interests.

## Authors' contributions

The idea for the article arose from the discussion of AJ and SS, with input from JHL. AJ and an experienced research assistant conducted the literature search, in consultation with SS. AJ and SS reviewed all abstracts. All authors abstracted data and assessed the quality of the articles. AJ completed the statistical analysis of the results and prepared the draft of the submission. All authors collaborated on reviewing and finalizing of the paper. SS and JHL controlled the decision to publish. All authors read and approved the final manuscript.

## Pre-publication history

The pre-publication history for this paper can be accessed here:



## References

[B1] McMurray JJV, Stewart S (2002). The burden of heart failure. Eur Heart J Sup.

[B2] Smith ER (2002). Heart failure – are we making progress?. Can J Cardiology.

[B3] Wollinsky FD, Overhage JM, Stump TE, Lubitz RM, Smith DM (1997). The risk of hospitalization for congestive heart failure among older adults. Med Care.

[B4] Wilson E (2001). Congestive heart failure: a national priority. Can J Cardiology.

[B5] Levy D, Kenchaiah S, Larson MG, Benjamin EJ, Kupka MJ, Ho KKL, Murabito JM, Vasan RS (2002). Long term trends in the incidence of and survival with heart failure. N Eng J Med.

[B6] McAllister FA, Lawson FME, Teo KK, Armstrong PW (2001). A systematic review of randomized trials of disease management programs in heart failure. Am J Med.

[B7] McAllister FA, Lawson FME, Teo KK, Armstrong PW (2001). Randomized trials of secondary prevention programmes in coronary heart disease: systematic review. BMJ.

[B8] Gwadry-Sridhar F, Flintoft V, Lee DS, Lee H, Guyatt GH (2004). A systematic review and meta-analysis of studies comparing readmission rates and mortality rates in patients with heart failure. Arch Intern Med.

[B9] Gonseth J, Guallar-Castillón P, Banegas JR, Rodriguez-Artalejo F (2004). The effectiveness of disease management programs in reducing hospital readmissions in older patients with heart failure: a systematic review and meta-analysis of published reports. Eur Heart J.

[B10] Gensichen J, Beyer M, Kuver C, Wang H, Gerlach FM (2004). [Case management for patients with congestive heart failure under ambulatory care – a critical review] [German]. Zeitschrift fur Arztliche Forbbildung und Qualitatssicherung.

[B11] Gibson PG, Powell H, Coughlan J, Wilson AJ, Abramson M, Haywood P, Bauman A, Hensley MJ, Walters EH (2005). Self-management education and regular practitioner review for adults with asthma. Cochrane Database of Systematic Reviews 2.

[B12] Deakin T, McShane CE, Cade JE, Williams RDRR (2005). Group based training for self-management strategies in people with type 2 diabetes mellitus. Cochrane Database of Systematic Reviews.

[B13] Monninkhof EM, van der Valk PDLPM, van der Palen J, van Herwaarden CLA, Partidge MR, Walters EH, Zielhuis GA (2005). Self-management education for chronic obstructive pulmonary disease. Cochrane Database of Systematic Reviews.

[B14] Health Canada (2005). Supporting self-care: The contribution of nurses and physicians: An exploratory study. http://www.hc-sc.gc.ca/hcs-sss/pubs/care-soins/1997-self-auto-contribut/literature_e.html.

[B15] Cochran WG (1954). Some methods for strengthening the common χ^2 ^tests. Biometrics.

[B16] Mantel N, Haenszel W (1959). Statistical aspects of the analysis of data from retrospective studies of disease. J Natl Cancer Inst.

[B17] Comprehensive Meta-analysis (2005). http://www.meta-analysis.com/html/v2preview.html.

[B18] Sethares K, Elliott K (2004). The effect of a tailored message intervention on heart failure readmission rates, quality of life, and benefit and barrier beliefs in persons with heart failure. Heart & Lung.

[B19] Koelling TD, Johnson ML, Cody RJ, Aaronson KD (2005). Discharge education improves clinical outcomes in patients with chronic heart failure. Circulation.

[B20] Krumholz HM, Amatruda J, Smith GL, Mattera JA, Roumanis SA, Radford MJ, Crombie P, Vaccarino V (2002). Randomized trial of an education and support intervention to prevent readmission of patients with heart failure. J Am Coll Cardiology.

[B21] Jaarsma T, Halfens R, Huijer Abu-Saad H, Dracup K, Gorgels T, van Ree J, Stappers J (1999). Effects of education and support on self-care and resource utilization in patients with heart failure. Eur Heart J.

[B22] Cline CM, Israelsson BY, Willenheimer RB, Broms K, Erhardt LR (1998). Cost effective management program for heart failure reduces hospitalization. Heart.

[B23] Ross SE, Moore LE, Earnest ME, Wittevrongel L, Chen-Tan L (2004). Providing a web-based online medical record with electronic communication capabilities to patients with congestive heart failure: randomized trial. J Med Internet Res.

[B24] Pincus T, Esther R, DeWalt DA, Callahan LF (1998). Social conditions and self-management are more powerful determinants of health than access to care. Annals Internal Med.

[B25] Louis AA, Turner T, Gretton M, Baksh A, Cleland JGF (2003). A systematic review of telemonitoring for the management of heart failure. Eur J Heart Fail.

